# Hyperbaric treatment for children with autism: a multicenter, randomized, double-blind, controlled trial

**DOI:** 10.1186/1471-2431-9-21

**Published:** 2009-03-13

**Authors:** Daniel A Rossignol, Lanier W Rossignol, Scott Smith, Cindy Schneider, Sally Logerquist, Anju Usman, Jim Neubrander, Eric M Madren, Gregg Hintz, Barry Grushkin, Elizabeth A Mumper

**Affiliations:** 1 International Child Development Resource Center, Melbourne, FL, USA; 2 Center for Autism Research and Education, Phoenix, AZ, USA; 3 True Health Medical Center, Naperville, IL, USA; 4 Edison, NJ, USA; 5 Princess Anne Medical Associates, Virginia Beach, VA, USA; 6 Therapeutic Pathways, East Troy, WI, USA; 7 Biognosys, Nanuet, NY, USA; 8 Rimland Center, Lynchburg, VA, USA

## Abstract

**Background:**

Several uncontrolled studies of hyperbaric treatment in children with autism have reported clinical improvements; however, this treatment has not been evaluated to date with a controlled study. We performed a multicenter, randomized, double-blind, controlled trial to assess the efficacy of hyperbaric treatment in children with autism.

**Methods:**

62 children with autism recruited from 6 centers, ages 2–7 years (mean 4.92 ± 1.21), were randomly assigned to 40 hourly treatments of either hyperbaric treatment at 1.3 atmosphere (atm) and 24% oxygen ("treatment group", n = 33) or slightly pressurized room air at 1.03 atm and 21% oxygen ("control group", n = 29). Outcome measures included Clinical Global Impression (CGI) scale, Aberrant Behavior Checklist (ABC), and Autism Treatment Evaluation Checklist (ATEC).

**Results:**

After 40 sessions, mean physician CGI scores significantly improved in the treatment group compared to controls in overall functioning (p = 0.0008), receptive language (p < 0.0001), social interaction (p = 0.0473), and eye contact (p = 0.0102); 9/30 children (30%) in the treatment group were rated as "very much improved" or "much improved" compared to 2/26 (8%) of controls (p = 0.0471); 24/30 (80%) in the treatment group improved compared to 10/26 (38%) of controls (p = 0.0024). Mean parental CGI scores significantly improved in the treatment group compared to controls in overall functioning (p = 0.0336), receptive language (p = 0.0168), and eye contact (p = 0.0322). On the ABC, significant improvements were observed in the treatment group in total score, irritability, stereotypy, hyperactivity, and speech (p < 0.03 for each), but not in the control group. In the treatment group compared to the control group, mean changes on the ABC total score and subscales were similar except a greater number of children improved in irritability (p = 0.0311). On the ATEC, sensory/cognitive awareness significantly improved (p = 0.0367) in the treatment group compared to the control group. Post-hoc analysis indicated that children over age 5 and children with lower initial autism severity had the most robust improvements. Hyperbaric treatment was safe and well-tolerated.

**Conclusion:**

Children with autism who received hyperbaric treatment at 1.3 atm and 24% oxygen for 40 hourly sessions had significant improvements in overall functioning, receptive language, social interaction, eye contact, and sensory/cognitive awareness compared to children who received slightly pressurized room air.

**Trial Registration:**

clinicaltrials.gov NCT00335790

## Background

Autistic Disorder (autism), along with Asperger syndrome and pervasive developmental disorder–not otherwise specified (PDD-NOS), comprise a spectrum of neurodevelopmental disorders (collectively termed autism spectrum disorders or ASD) that are characterized by restrictive and repetitive behaviors along with impairments in communication and social interaction [1]. The number of children diagnosed with ASD has increased over the last decade [2-4] and ASD currently affects as many as 1 out of 150 individuals in the United States (U.S.) [5]. ASD is generally considered a "static" neurological disorder [6] without any known cure. The use of hyperbaric treatment in children with ASD has increased in recent years [7] and traditionally involves inhaling up to 100% oxygen at a pressure greater than one atmosphere (atm) in a pressurized chamber [8]. Most typical indications for hyperbaric treatment involve the use of hyperbaric pressures above 2.0 atm. Higher atmospheric pressures are generally required to treat conditions such as carbon monoxide poisoning and to improve wound healing [8,9]. However, improvements have been observed via treatments with 95–100% oxygen and hyperbaric pressures of 1.5–2.0 atm for some chronic neurological conditions, including autism [7], fetal alcohol syndrome [10], cerebral palsy [11,12], and chronic or traumatic brain injury [13-16]. Furthermore, improvements in some of these conditions, including autism [7,17] and cerebral palsy [12], have been observed with the use of hyperbaric pressures of 1.3 atm and oxygen levels of 21–24%. In one study, significant improvements were observed in children with autism with the use of hyperbaric treatment at both 1.5 atm/100% oxygen and 1.3 atm/24% oxygen; neither hyperbaric protocol worsened markers of oxidative stress and both reduced C-reactive protein (a marker of inflammation) [7]. Rationales for the use of hyperbaric treatment in autism include decreasing inflammation [18-20], improving cerebral hypoperfusion [21,22], and modulating immune dysregulation [23-25], all reported as problems in some individuals with autism [26-34]. Several case reports [21,22] and three uncontrolled studies enrolling between 6 and 18 children with autism [7,17,35] have reported clinical improvements with hyperbaric treatment at 1.3 atm. However, to our knowledge, the efficacy of hyperbaric treatment in children with autism has not been evaluated to date with a controlled study. Given this background, we decided to study the effects of hyperbaric treatment in children with autism using 1.3 atm and 24% oxygen compared to near-placebo hyperbaric conditions (slightly pressurized room air at 1.03 atm and 21% oxygen).

Hyperbaric treatment for children is generally regarded as safe, even at pressures of 2.0 atm and 100% oxygen for two hours per day [36]. In descending order, the most common side effects observed during hyperbaric treatment are barotrauma (2% incidence), sinus squeeze, serous otitis, claustrophobia, reversible myopia, and new onset seizure (which occurs in 1–3 per 10,000 treatments) [8]. In children with autism, the use of hyperbaric treatment using pressures up to 1.5 atm and 100% oxygen has been shown to be safe and well-tolerated [7,17].

## Methods

### Study Design

This was a prospective, randomized, double-blind, controlled trial involving treatment in parallel groups for 4 weeks. Active treatment was hyperbaric treatment at 1.3 atm and 24% oxygen for 40 sessions lasting 1 hour each at pressure ("treatment group"), whereas the control treatment consisted of slightly pressurized room air at 1.03 atm and 21% oxygen for 40 sessions lasting 1 hour each at pressure ("control group"). Comparison of the clinical effects of parallel treatments for 4 weeks was the primary objective of this study. The number of treatments (40 sessions) and the overall treatment period (4 weeks) were chosen because these were previously shown to be safe in two other studies of hyperbaric treatment in children with autism [7,17].

### Participants: Eligibility Criteria and Recruitment

**Figure 1:**
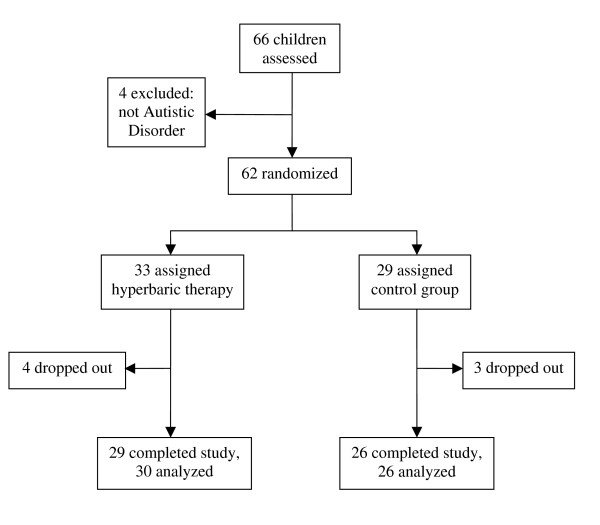
**Consolidated Standards of Reporting Trials (CONSORT) flow diagram**.

This study was approved by the Liberty Institutional Review Board and enrolled children, 2 to 7 years of age, who had a diagnosis of Autistic Disorder and had not previously received any type of hyperbaric treatment. All children met the DSM-IV criteria for Autistic Disorder [1] and this diagnosis was also corroborated by psychologists using the Autism Diagnostic Interview–Revised (ADI-R) and the Autism Diagnostic Observation Schedule (ADOS). Children with PDD-NOS, Asperger syndrome, seizure disorder, current ear infection, uncontrolled asthma, inability to equalize ear pressure, fragile X syndrome, and ongoing treatment with chelation medication were excluded from participation in this study. Written informed consent was obtained from the parents and, when possible, the child. Sixty-six children were evaluated for inclusion in the study from six clinics throughout the U.S. Four children were excluded from participation because the diagnosis of Autistic Disorder could not be corroborated by ADI-R and ADOS. Therefore, the recruitment process yielded 62 eligible participants, who were randomized as depicted in Figure 1.

### Interventions

The active treatment was hyperbaric treatment at 1.3 atm and 24% oxygen in a monoplace hyperbaric chamber for 60 minutes at this pressure per session (this length of time did not include approximately 10–15 minutes for pressurization and depressurization). Oxygen flowing at 10 liters per minute from an oxygen concentrator was mixed with room air and pumped into the chamber following a protocol previously described [7]. This resulted in a final chamber oxygen concentration of approximately 24% as measured by an oxygen monitor. This treatment was given twice a day separated by a minimum of 4 hours, 5 days per week, for 4 consecutive weeks, for a total of 40 treatments per child.

Control treatment consisted of slightly pressurized room air (1.03 atm and 21% oxygen) in a monoplace hyperbaric chamber for 60 minutes at this pressure per session (this length of time did not include approximately 10–15 minutes for pressurization and depressurization). This treatment was given twice a day separated by a minimum of 4 hours, 5 days per week, for 4 consecutive weeks, for a total of 40 treatments per child. For blinding purposes, participants underwent a brief compression to 1.1 atm at the beginning of each treatment. The chamber was then slowly decompressed from 1.1 to 1.03 atm where the pressure stayed for the remainder of the treatment. No oxygen was added to the chamber and thus the chamber was pressurized with room air (approximately 21% oxygen). The children in the control group remained in the chamber for the same length of time as children in the treatment group. At the end of each treatment, the pressure was slowly increased to 1.1 atm over about 5 minutes and then the chamber was depressurized. Procedures were developed and applied to mimic, for the control group, the experience of hyperbaric treatment at 1.3 atm, and thereby to keep participants and parents unaware of the nature of the intervention. These procedures included covering control switches, inflating and deflating the chambers to simulate pressure changes, and masking the sounds from the chambers. To further mask the group assignments, the equipment (including chambers) used for the control group was indistinguishable from the equipment used for the treatment group. Moreover, the same type of equipment was used at each study site. A pressure of 1.03 atm (with increases to 1.1 atm for several minutes at the beginning and at the end of the treatment) was chosen for the control group because this pressure represented the lowest that could be applied and still effectively simulate hyperbaric treatment at 1.3 atm. To verify its effectiveness, prior to beginning the study, this pressure protocol was tested in six adult individuals who were randomly and repeatedly exposed to both the treatment group pressure (1.3 atm) and the control group pressure (1.03 atm with short increases to 1.1 atm) and none of these individuals were able to reliably distinguish between the two pressures. At each study site, the primary investigator (DAR) visited and trained each hyperbaric technician to ensure that the same protocol was followed to minimize variances between study sites. An analysis performed after the study was finished demonstrated no significant differences (p = ns) between the six study sites for age, initial autism severity, and initial and final scores on all of the scales used in this study (this analysis can be found in the results section).

Initial screening for this study included medical history taking and a physical examination by one of the study physicians. This included examination of the ears and tympanic membranes. Throughout each treatment, children were closely monitored by a hyperbaric technician for any signs of ear pain or other problems, and parents were instructed on how to recognize ear pain in their child. In both groups, in order to facilitate treatments, a parent or primary caretaker accompanied the child into the chamber as it was deemed that the children were too young to enter and remain in the chamber alone for the duration of each treatment. Children finishing more than one full session were included in the final analysis in an intention-to-treat manner. Daily treatment logbooks for each child were maintained by the hyperbaric technician and any side effects during treatment were recorded. At the end of the study, all children assigned to the control group were offered 40 hyperbaric treatments at the treatment pressure (1.3 atm and 24% oxygen) if the parents desired (all parents chose this option; treatments were provided without charge). During the study period, children in both groups were not allowed to begin any new therapies or stop any current therapies, including medications and nutritional supplementation. At the onset of the study, the use of nutritional supplements, medications, and applied behavioral analysis (ABA) therapy was similar in both the treatment and control groups (p = ns), see Table 1.

**Table 1:** Initial characteristics of children in the treatment and control groups

	Treatment	Control	Comparison between groups (p-value)
Age	4.97 ± 1.29	4.86 ± 1.13	0.7288*

Male	30/33	22/29	0.1672**

Use of nutritional supplements	23/33	20/29	0.9999**

Use of medications	16/33	10/29	0.3915**

Applied Behavioral Analysis Therapy	15/33	11/29	0.733**

ABC Total Score	55.2 ± 28.7	53.3 ± 24.0	0.7843*

ABC Irritability	13.2 ± 9.5	12.2 ± 7.9	0.6714*

ABC Social Withdrawal	10.5 ± 6.9	11.2 ± 6.9	0.7048*

ABC Stereotypy	7.5 ± 4.9	6.2 ± 4.7	0.3205*

ABC Hyperactivity	20.7 ± 9.9	20.1 ± 8.2	0.8279*

ABC Speech	3.4 ± 3.1	3.6 ± 3.6	0.8567*

ATEC Total Score	75.3 ± 19.5	75.6 ± 21.0	0.9592*

ATEC Speech/Language/Communication	16.3 ± 5.0	15.9 ± 6.1	0.7958*

ATEC Sociability	17.4 ± 6.6	17.8 ± 6.2	0.849*

ATEC Sensory/Cognitive Awareness	18.1 ± 5.2	19.6 ± 5.6	0.3676*

ATEC Health/Physical/Behavior	23.5 ± 11.5	22.4 ± 8.3	0.72*

** Chi-square test with Yates' correction or Fisher's exact test.

* Student's t test

### Randomization and Allocation

From the 62 children who were enrolled in the study, 33 were randomly allocated to the treatment group and 29 were randomly allocated to the control group. To achieve this allocation, a random allocation sequence (1:1) was generated and stratified on both the participant's group (treatment or control) and center, and this sequence was equilibrated every eight patients. The allocation sequence remained concealed to all investigators, study participants, parents, nursing staff, and all other clinic staff. The only person at each center who was aware of the group assignment for each particular child was the hyperbaric technician, who had no input in the evaluation (outcome measures) of the child. The hyperbaric technician was specifically instructed not to discuss the treatment nature or group assignments with anyone else in the clinic, including participants, parents, psychologists, and physicians. It was not possible to blind the hyperbaric technician due to the nature of the study (the technician had to know the group assignment in order to adjust the chamber to the correct pressure for treatment). However, all individuals involved in evaluating the child (parents, physicians, and psychologists) remained blinded to the group assignment for each child throughout the entire study period. In hyperbaric treatment studies, the study is considered double-blinded if the study participants and the evaluators of outcome measures are both blinded to group assignment (as they were in this study), even though the hyperbaric technician is aware of the assignment [37,38]. After the study was completed, parents in both groups were surveyed to determine the effectiveness of the blinding process, and there was no significant difference between groups in their ability to determine which group their child had been assigned (p = ns).

### Outcome measures

The primary outcome measures were changes compared to baseline observed after 4 weeks (40 sessions) of treatment, in parallel groups, on (1) Aberrant Behavior Checklist–Community (ABC) total score and subscale scores, based on the parent's or primary caretaker's rating, (2) Autism Treatment Evaluation Checklist (ATEC) total score and subscale scores, based on the parent's or primary caretaker's rating, and (3) Clinical Global Impression–Improvement (CGI) scale for changes in overall functioning and subscales, as rated by the parent or primary caretaker and also as rated separately by the treating physician (without knowledge of the parental ratings).

Previous studies of hyperbaric treatment in children with autism have utilized the ABC and/or ATEC [7,17]. The ABC is a 58-item questionnaire that assesses communication, reciprocal social interaction, play, and stereotypical behaviors [39]. It is used to evaluate the effectiveness of medications and other therapeutic interventions and is scored from 0 ("not at all a problem") to 3 ("problem is severe in degree"). For this study, a total score was calculated as well as scores in 5 subscales: irritability, social withdrawal (also termed lethargy), stereotypy, hyperactivity, and inappropriate speech. The ABC was administered immediately prior to beginning the study (to determine baseline scores) and immediately after finishing 40 sessions. Lower scores on the ABC indicate lower autism severity.

The ATEC is a questionnaire developed by the Autism Research Institute to evaluate treatment efficacy in individuals with autism. It consists of four subscales: Speech/Language/Communication, Sociability, Sensory/Cognitive Awareness, and Health/Physical/Behavior. The scores are weighted according to the response and the corresponding subscale. The higher the subscale and total scores, the more impaired the subject. A split-half reliability analysis on 1,358 checklists indicated high internal consistency among the questions within each subscale [40]. The ATEC is designed to allow evaluators to assess outcomes of certain treatments commonly used in individuals with autism. In this study, scores were calculated for the total score and the four separate subscales. The ATEC was administered immediately prior to beginning the study (to determine baseline scores) and immediately after finishing 40 sessions. Due to an administration error, the baseline ATEC was not performed at one of the study centers, and thus data was available for analysis for 23 children in the treatment group and 21 children in the control group.

Scores for the CGI scale were obtained immediately after 40 sessions. The CGI scale gives an impression of global changes in certain areas for each child compared to baseline [41]. A total score for change in overall functioning was rated by a parent or primary caregiver and separately by the treating physician (the same physician who initially evaluated the child) using the following ratings: 1 ("very much improved"), 2 ("much improved"), 3 ("minimally improved"), 4 ("no change"), 5 ("minimally worse"), 6 ("much worse"), and 7 ("very much worse"). Children who received a score of "very much improved" or "much improved" on the physician CGI overall functioning score were considered to be "good responders" to treatment. Data was also collected from parents and physicians as to whether or not there were improvements in the following CGI subscales: receptive language, expressive language, sleep pattern, attention span, activity level, bowel movement pattern, self-stimulatory behavior, social awareness/alertness, social interaction, play skills, self-injurious behavior, eye contact, mood, anxiety level, aggression, general health, gross motor skills, and fine motor skills.

### Analysis

All data were prospectively collected and analyzed using StatsDirect statistical software (version 2.7.2) and are presented as mean ± SEM (standard error of the mean). Data analysis was based on an intention-to-treat approach on all participants who finished more than one treatment or control session.

#### Power Calculations

Because this was the first controlled study of its kind, power calculations were based on the closest comparable study that had outcome data available at the time of this study design [17]. Analysis of this data demonstrated a medium to large effect size, depending on the scale examined (Cohen's d = 0.44 to 0.77) [42]. Using the most conservative effect size (d = 0.44), a power calculation using G*Power 3 [43] indicated that a total sample of 43 children would achieve a power of 80% with alpha set at 0.05 (two-tailed).

#### Planned Comparisons

Planned group comparisons were performed on the primary outcome measures. The normal distribution of data was checked using the Kolmogorov-Smirnov test. In parameters with normal distribution, comparisons were analyzed using the Student's t test. When normality was not present and equal variance could not be assumed, nonparametric tests (Mann-Whitney and Wilcoxon signed rank tests) were used. The Pearson's chi-square (χ^2^) test with Yates' correction or the Fisher's exact test (when subgroups contained less than 10 children) was applied to assess differences in the percentage of children responding to treatment in each group. In all analyses, a p-value less than 0.05 (two-tailed) was considered significant.

## Results

### Study Sample

The flow of participants throughout the study is depicted in Figure 1. This study consisted of 52 boys and 10 girls, which is consistent with the male/female ratio observed in children with autism [44]. The mean age of all children was 4.92 ± 1.21 years and was similar (p = ns) in the treatment group (4.97 ± 1.29 years) and the control group (4.86 ± 1.13 years), see Table 1. There were more girls in the control group compared to the treatment group, but this difference was not significant (p = ns). Initial ABC and ATEC scores were similar in both groups (p = ns). At the onset of this study, the use of nutritional supplements, medications, and applied behavioral analysis (ABA) therapy was similar in both groups (p = ns), see Table 1. One-way analysis of variance (ANOVA) demonstrated no significant differences (p = ns) between the six centers that participated in this study for: age, initial autism severity, initial ABC total scores, final ABC total scores, initial ATEC total scores, final ATEC total scores, physician CGI scores, or parental CGI scores.

Attrition rates during the study were low (see Figure 1). In the treatment group, two children dropped out of the study prior to beginning any treatments due to an illness (one with otitis media, the other with bronchitis). Another child dropped out before finishing one full treatment due to anxiety in both the child and the parent. Finally, one child was removed from the study after nine sessions because asthma symptoms worsened (neither the parents nor the treating physician felt that the hyperbaric treatments contributed to the increased asthma symptoms, but the child was removed from the study as a precaution); this child's scores performed at time of drop-out showed mild improvements in behavior (as separately ranked by both the physician and the parents) and these scores were included in the intention-to-treat analysis. The inclusion or exclusion of this child's scores had no significant effect on the statistical analysis. The remaining 29 children completed all 40 hyperbaric treatment sessions at 1.3 atm and 24% oxygen. Therefore, data from 30 children were analyzed in the treatment group.

In the control group, two children dropped out of the study prior to beginning any treatments (one because of a death in the family, the other because of the time commitment). One child dropped out prior to finishing one full treatment due to parental claustrophobia. The remaining 26 children finished all 40 sessions at 1.03 atm and 21% oxygen.

### Outcome Measures

#### Physician Clinical Global Impression (CGI) Scale

**Figure 2:**
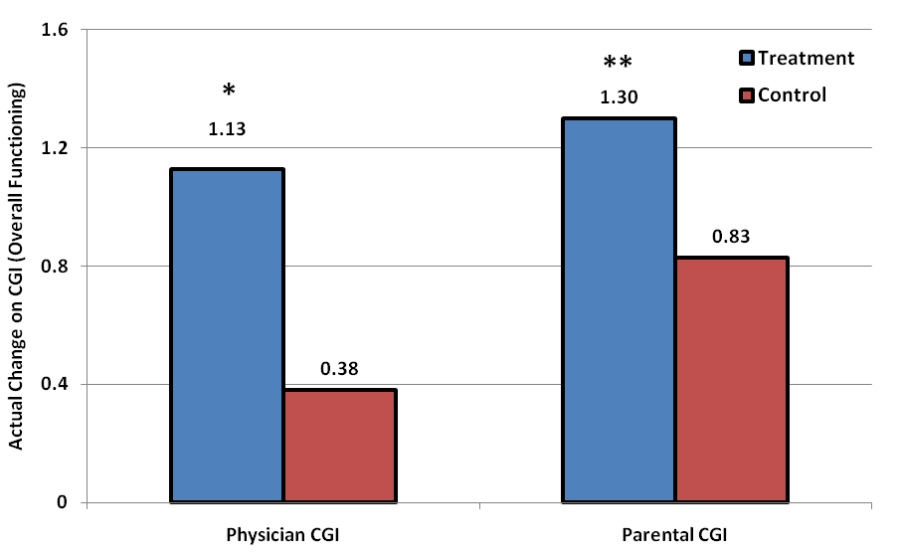
**Absolute change compared to baseline on the mean CGI overall functioning score in the treatment and control groups as rated separately by physicians and parents**. * p < 0.001; ** p < 0.05.

After 40 sessions, the mean physician CGI score for change in overall functioning compared to baseline significantly improved (p = 0.0008) by 1.13 points in the treatment group (2.87 ± 0.78, score of 4 = "no change") compared to 0.38 points in the control group (3.62 ± 0.75), see Figure 2. Furthermore, 9/30 (30%) children in the treatment group had a "very much improved" or "much improved" rating compared to 2/26 (7.7%) in the control group (p = 0.0471). An improvement on the CGI scale (score of 1, 2, or 3) was noted in 24/30 (80%) children in the treatment group compared to 10/26 (38%) in the control group (p = 0.0024). Conversely, 16/26 (62%) children in the control group had a "no change" or "minimally worse" score (CGI score of 4 or 5) compared to 6/30 (20%, all 6 had a score of 4) in the treatment group (p = 0.0024). In the control group, two children received a score of 5 ("minimally worse"), whereas none received this score in the treatment group (p = 0.211). No child received a score worse than 5 in either group. Examination of the physician CGI subscales demonstrated that more children improved in the treatment group compared to the control group in receptive language (p < 0.0001), social interaction (p = 0.0473), and eye contact (p = 0.0102); a trend towards improvement was also observed in activity level (p = 0.0545).

#### Parental Clinical Global Impression (CGI) Scale

The mean parental CGI score for change in overall functioning compared to baseline significantly improved (p = 0.0336) by 1.30 points in the treatment group (2.70 ± 0.81, score of 4 = "no change") compared to 0.83 points in the control group (3.17 ± 0.73), see Figure 2. A "very much improved" or "much improved" rating was observed in 9/30 (30%) children in the treatment group compared to 4/26 (15%) in the control group (p = 0.2238). Furthermore, 27/30 (90%) children had an improvement on the CGI scale (score of 1, 2, or 3) in the treatment group compared to 19/26 (73%) in the control group (p = 0.1616). A score of "no change" or "minimally worse" (CGI score of 4 or 5) was reported in 3/30 (10%, all scored 4) in the treatment group versus 7/26 (27%) in the control group (p = 0.1616). One child received a score of 5 ("minimally worse") in the control group compared to none in the treatment group (p = 0.4643). No child received a score worse than 5 in either group. Examination of the parental CGI subscales demonstrated that more children improved in the treatment group compared to the control group in receptive language (p = 0.0168) and eye contact (p = 0.0322).

Examination of the mean CGI score for change in overall functioning in the treatment group as rated by the physicians compared to the parental ratings demonstrated that there was no significant difference (p = 0.4716). A significant correlation existed between the physician and parental CGI scales for the treatment group (r = 0.60, p = 0.0005). However, parents of children in the control group were significantly more likely to rate an improvement on the CGI score for change in overall functioning than were physicians (p = 0.0245) and therefore the correlation between the physician and parental CGI scales was not significant (r = 0.27, p = 0.1819).

#### Aberrant Behavior Checklist (ABC) Scores

**Figure 3:**
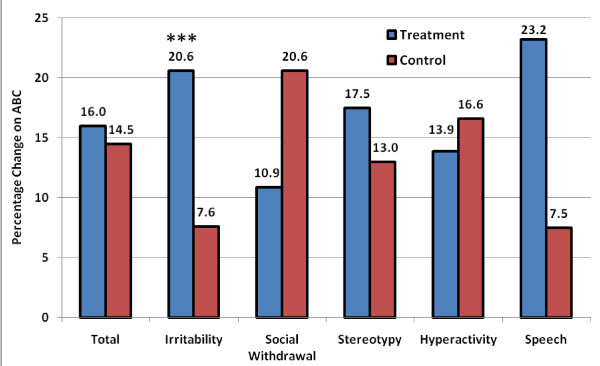
**Changes compared to baseline on the ABC total score and subscales (percentage change) in the treatment and control groups**. *** p < 0.1.

In the treatment group, the ABC total score significantly improved after 40 sessions (p = 0.0118), see Additional file 1. Improvements in ABC subscales were also observed in the treatment group for irritability (p = 0.0147), stereotypy (p = 0.0124), hyperactivity (p = 0.0211), and speech (p = 0.0155). No significant improvements were observed in the control group on the ABC total score or any of the ABC subscales (p = ns). Analysis of changes in the ABC total score and subscale scores between the treatment and control groups demonstrated no significant changes (p = ns), although there was a trend towards improvement in the treatment group for irritability (p = 0.0976, see Figure 3) and 20/30 (67%) children in the treatment group had an improvement in irritability compared to 9/26 (35%) in the control group (p = 0.0311).

#### Autism Treatment Evaluation Checklist (ATEC) Scale

**Figure 4:**
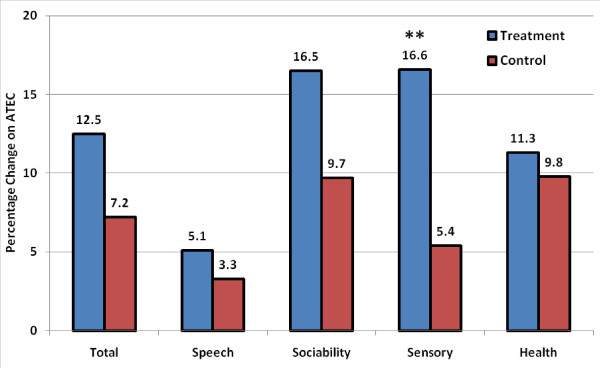
**Changes compared to baseline on the ATEC total score and subscales (percentage change) in the treatment and control groups**. ** p < 0.05.

In the treatment group, significant improvements were observed on the ATEC scale in total score (p = 0.002), sociability (p = 0.0009), sensory/cognitive awareness (p = 0.0017), and health/physical/behavior (p = 0.0446), see Additional file 2. In the control group, ATEC improvements were found in total score (p = 0.0385) and sociability (p = 0.0134). Analysis of changes in ATEC total score and subscale scores between the treatment and control groups showed a significant improvement in sensory/cognitive awareness in the treatment group (p = 0.0367), see Figure 4. Non-significant improvements in the treatment group compared to the control group were observed in the other ATEC subscales (p = ns).

#### Analysis by age and autism severity

Because we had previously observed that both younger children and children who had higher initial autism severity improved more robustly with hyperbaric treatment at 1.3 atm [7,17], two separate sub-analyses of the effects of age and initial autism severity on the outcome scales used in this study were performed to determine if a subgroup could be identified that had a better response to hyperbaric treatment.

##### Age

Post-hoc analysis of children in the treatment group demonstrated a better improvement on the ABC total score in children who were over age 5 compared to those age 5 and under (p = 0.0482). Comparison of children who were over age 5 in the treatment and control groups demonstrated that children in the treatment group had significantly better improvements on the ABC in irritability (p = 0.0149), social withdrawal (p = 0.0086), and stereotypy (p = 0.0434). There was no significant difference in ABC scores between the treatment and control groups for children age 5 and younger (p = ns). When examining the ATEC scale, comparison of children who were over age 5 between the treatment and control groups demonstrated that children in the treatment group had significantly better improvements in sociability (p = 0.0095) and sensory/cognitive awareness (p = 0.0384). No significant difference between the two groups was observed for children age 5 and younger on the ATEC scale (p = ns). No significant age effect (p = ns) was observed between the treatment and control groups on the parental or physician CGI scales.

##### Autism severity

Post-hoc analysis of children in the treatment group demonstrated that those who had an initial ADOS score below the 50^th ^percentile for all children (less initial autism severity) had similar improvements in ABC total score and subscales compared to children with an initial ADOS score above the 50^th ^percentile (p = ns). However, comparison of children in the treatment group and the control group who had an initial ADOS score below the 50^th ^percentile (less autism severity) demonstrated that the children in the treatment group had significantly better improvements in ABC irritability (p = 0.0348) and ABC stereotypy (p = 0.0359). There was no significant difference in ABC scores between the treatment and control groups for children with an initial ADOS score above the 50^th ^percentile (p = ns). When examining the ATEC scale, comparison of children in the treatment group and the control group with an initial ADOS score below the 50^th ^percentile demonstrated a significantly better improvement in the treatment group in sociability (p = 0.0333). No significant difference between groups was observed for children with an initial ADOS score above the 50^th ^percentile (p = ns). No significant effect (p = ns) was observed for autism severity between the treatment and control groups on the parental or physician CGI scales.

### Adverse events and tolerance

Hyperbaric treatment in this study was safe and well-tolerated. In the treatment group, one child developed both urinary frequency (urinalysis was normal) and a skin rash that the treating physician thought was yeast-related. As previously described, one child had worsening of asthma symptoms after nine treatment sessions and was removed from the study, and another child had anxiety and dropped out of the study before finishing one full treatment. None of the children in the treatment group received a score worse than 4 ("no change") on the physician or parental CGI for change in overall functioning. In the control group, one child developed abdominal distension and diarrhea during the study, but was able to complete the study. Another child in the control group had worsening of eczema during the study. No other adverse events including barotrauma or seizures were observed in either group.

## Discussion

To our knowledge, this trial represents the first controlled study of hyperbaric treatment in children with autism. Previous studies examining this treatment in autism have described improvements that could have been due, in part, to a participation (placebo) effect. The results of uncontrolled studies in autism should be interpreted with caution, especially since some randomized, double-blind, placebo-controlled studies in individuals with autism have reported relatively high improvement rates in the placebo group. For example, one prospective study comparing a single dose of IV secretin to a placebo found that 30% of the children receiving the placebo had a significant improvement immediately after the infusion [45]. Another prospective study comparing daily treatment with amantadine to a placebo over a 4-week period found a mean placebo response rate of 37% [46]. In the current study, 80% of children in the hyperbaric treatment group had an improvement on the CGI scale for change in overall functioning as rated by blinded physicians; however, 38% of children in the control group were also rated as improved. This 38% improvement rate in the control group may have occurred because these children received a very low level of hyperbaric pressure (1.03 atm with short increases to 1.1 atm), and therefore, strictly speaking, this pressure did not represent a true placebo-control group. Hyperbaric pressure as low as 20 mmHg (approximately 1.03 atm) has been shown to decrease *in vitro *pro-inflammatory cytokine release (including IL-1beta) from human monocytes and macrophages [47]. Some children with ASD have elevations in certain pro-inflammatory cytokines, including IL-1beta [32,48]. Therefore, some of the improvements observed in the control group could have been due to the slight hyperbaric pressure received. Because the control group experienced pressure conditions closer to those of the treatment group than a true placebo (e.g., 1.00 atm and 21% oxygen) would have provided, the difference in clinical outcomes between the treatment and control groups may have been less significant than what would have been observed with a placebo. However, a true placebo could not have been used with this study design because some degree of hyperbaric pressure was needed to mimic hyperbaric treatment at 1.3 atm, otherwise blinding of the group assignment would have not been possible. A pressure of 1.03 atm (with short increases to 1.1 atm) was chosen for the control group because testing performed prior to the study indicated that this pressure was the lowest that could be given and still effectively simulate, from the perspective of the blinded parents and children, hyperbaric treatment at 1.3 atm. The blinding procedure in this study appeared to be adequate because there was no significant difference between the two groups in the ability of parents to correctly guess the group assignment of their child. Furthermore, 73% of parents of children in the control group rated their child as improved on the CGI scale which also suggests that the blinding procedure was adequate, because if parents thought that their child was in the control group, they probably would have been less likely to rate an improvement after treatment. In the hyperbaric treatment group, parental CGI scores significantly correlated with physician CGI scores (r = 0.60, p = 0.0005) which strengthens the CGI results in this group. In the control group, the parents were significantly more likely to rate their child as improved on the CGI scale compared to the physicians (p = 0.0245) and therefore the parental and physician CGI scales did not significantly correlate (r = 0.27, p = 0.1819). This finding further suggests that the blinding procedure was adequate in this study and also demonstrates evidence of a participation effect in the control group.

In this trial, the use of hyperbaric treatment at 1.3 atm was well-tolerated and confirmed previous reports of safety. This study also demonstrated clinical improvements that were similar to previous uncontrolled studies of hyperbaric treatment in children with autism [7,17,21,22,35]. The findings of this study are significantly strengthened because of the presence of a control group which previous hyperbaric treatment studies in autism lacked, and also because of the use of six separate centers which should have minimized potential bias, especially since there were no significant differences between study sites in age, initial autism severity, and initial and final scores on all of the scales used in this study. In this current trial, significant improvements were observed in several domains with the use of hyperbaric treatment at 1.3 atm and 24% oxygen compared to slightly pressurized room air, including overall functioning, receptive language, social interaction, eye contact, and sensory/cognitive awareness. The reason for these different areas of improvement is not clear. The mechanism of action of hyperbaric treatment in autism is not entirely known, although it may act by diminishing gastrointestinal and cerebral inflammation and by improving immune dysregulation and cerebral hypoperfusion [24]. Multiple studies have reported that these problems are relatively common in children with autism [26-34].

Cerebral hypoperfusion, especially of the temporal lobes, is a very common finding in children with autism compared to typically-developing children, affecting up to 75% [28,49]. This hypoperfusion is an indirect measure of diminished brain activity [28] because cerebral blood flow is normally tightly coupled to brain metabolic rate and function [50,51]. Several studies have reported that the anatomical location of cerebral hypoperfusion significantly correlates with certain autistic behaviors [24]. For example, in a study of 30 individuals with autism compared to 14 non-autistic individuals, hypoperfusion of the thalamus as measured by Single Photon Emission Computed Tomography (SPECT) was observed in the autism group and significantly correlated (r = 0.42, p < 0.01) with repetitive behaviors and unusual sensory interests [52]. In another SPECT study of 23 children with autism compared to 26 non-autistic children, hypoperfusion of the right medial temporal lobes was found in the autism group and was correlated with obsessive desire for sameness (p < 0.001), and hypoperfusion of the medial prefrontal cortex and anterior cingulate gyrus was associated with impairments in social interaction and communication (p < 0.001) [27]. Furthermore, two SPECT studies in individuals with autism have reported that cerebral hypoperfusion significantly worsens with increasing age [53,54]. In one of these studies, hypoperfusion of brain areas that controlled speech (left temporal lobe and frontal areas) significantly worsened with increasing age (p < 0.001) and was associated with deficits in language formation and "subsequently prevent [ed] development of true verbal fluency and development in the temporal and frontal areas associated with speech and communication" [54]. Furthermore, in another study of 45 children with autism, children with the highest degree of left temporal lobe hypoperfusion, as measured by Positron Emission Tomography (PET), also had the most severe autistic behavior [55].

The cause of cerebral hypoperfusion in children with autism is not known. Several studies have described apparent vascular-associated cerebral inflammation in children with autism compared to controls including perivascular macrophage and microglia accumulation in post-mortem autistic brain samples [33] as well as the presence of serum IgM and IgG autoantibodies that bind to small blood vessels in the brain in about 30% of children [26,56]. These findings could be consistent with a cerebral vasculitis [24]. Elevated urinary levels of 8-isoprostane-F2α have also been reported in some children with autism [57]. In some studies, this isoprostane elevation has been shown to cause *in vivo *vasoconstriction and increase the aggregation of platelets [58]. Furthermore, elevations in 2,3-dinor-thromboxane B_2 _(associated with increased platelet activation) and 6-keto-prostaglandin F_1α _(a marker of endothelium activation) have been described in some children with autism [59]. These inflammatory-related findings could contribute to the cerebral hypoperfusion described in autism [24].

Cerebral hypoperfusion is associated with hypoxia [24] and several studies in children with ASD have reported evidence of cerebral hypoxia, as measured by a reduction in brain Bcl-2 and an increase in brain p53 [60-63]. Elevated p53 is induced by hypoxia [64] and a decrease in Bcl-2 is associated with increased apoptosis provoked by hypoxia [65]. Hypoxia leads to higher brain concentrations of hypoxia-inducible factor 1α (HIF-1α) [66]. An increase in HIF-1α causes an increase in inflammation, including redness and swelling of tissues, and the attraction of lymphocytes [66]. HIF-1α is essential for inflammation mediated by myeloid cells [67]. In fact, in one study, rats that were null for HIF-1α demonstrated almost complete inhibition of the inflammatory response [68]. HIF-1α is responsible for angiogenesis that is secondary to hypoxia [68,69] and also induces Vascular Endothelial Growth Factor (VEGF), which increases the permeability of blood vessels [66] and causes tissue edema. Evidence of cerebral edema in 19 children with autism compared to 20 typically-developing children was suggested by one recent T2-magnetic resonance imaging (MRI) study [70]. This edema can lead to increased interstitial space between cells [71] and cause an increase in the distance that oxygen must diffuse from blood vessels to reach brain cells and can thus lead to cellular hypoxia [72]. Inflammation is also associated with blood-brain barrier disturbances which can further increase cerebral edema [24]. Chronic inflammation is commonly associated with the infiltration of polymorphonuclear neutrophils and other immune cells, along with the cytokines that are released by these cells. This causes an increase in local oxygen usage due to the elevated oxygen requirements created by these newly infiltrated cells. Yet, at the same time, inflammation causes reduced oxygen extraction by normal cells [73]. For instance, in one study, elevated markers of inflammation (including IL-6, tumor necrosis factor receptors 1 and 2, and high-sensitivity C-reactive protein) were significantly correlated with decreased maximum oxygen uptake at peak exercise (VO_2_max) in patients with known or suspected coronary artery disease [74]. Therefore, inflammation prevents maximal uptake of oxygen by cells. Inflammation also increases oxidative stress and can cause neutrophils to become more adherent and attach to vessel walls [75]. This infiltration and increased adherence of inflammatory cells can contribute to brain injury by decreasing microvascular blood flow, causing thrombosis, and increasing the production of free radicals [76]. Hyperbaric treatment can overcome the effects of cerebral hypoperfusion and hypoxia by: increasing the plasma oxygen tension which transfers more oxygen into tissue, including the brain [77,78], decreasing cerebral edema [79], inhibiting the expression of HIF-1α and its target genes [80], and by causing angiogenesis over time [18].

Several case reports in children with autism have described improved cerebral perfusion after hyperbaric treatment at 1.3 atm, as measured by post-hyperbaric treatment SPECT scans compared to pre-hyperbaric SPECT scans [21,22]. If the hypoperfusion in children with autism is related to cerebral inflammation, then hyperbaric treatment could potentially improve cerebral perfusion by decreasing this inflammation [24]. Hyperbaric treatment possesses strong anti-inflammatory properties [18-20] and has been shown to significantly decrease neuroinflammation [81] as well as cerebral edema and blood-brain barrier damage in animal models [79]. At 1.3 atm, hyperbaric treatment decreased a marker of inflammation (C-reactive protein) in one study of children with autism [7]. It is unknown if any of the improvements observed in this study were mediated through an improvement in cerebral hypoperfusion and/or a decrease in cerebral inflammation as this study was not designed to examine these possibilities. However, since cerebral hypoperfusion is relatively common [28,49] and can be diffuse in location in children with autism [82,83], and the anatomical location of hypoperfusion significantly correlates with certain autistic behaviors [27,52,54], then improving hypoperfused brain areas with hyperbaric treatment could account for the different areas of improvement observed in this study.

Our previous studies suggested children who were younger and those who had higher initial autism severity responded more robustly to hyperbaric treatment [7,17]. However, these studies were small and uncontrolled, and thus we analyzed these two parameters (age and autism severity) in this study with a post-hoc analysis. An interesting finding from this current study was that children who were over age 5 had significantly better improvements on the ABC total score with hyperbaric treatment at 1.3 atm compared to younger children (p = 0.0482). Given the fact that older children with autism generally have a higher degree of cerebral hypoperfusion compared to younger children [53,54] and that hyperbaric treatment can improve cerebral hypoperfusion [21,22], these factors could have accounted for the age findings observed in this study. Additional studies examining the use of hyperbaric treatment in children with autism that also incorporate SPECT or PET scans to measure changes in cerebral blood flow might be helpful in further delineating these possibilities. Moreover, children who had lower initial autism severity also had the most improvements with hyperbaric treatment in this study. The reason for this finding is not known, but may be due to greater levels of oxidative stress and other metabolic problems recently described in children with higher autism severity compared to those with lower severity [84].

Because this study was not designed to measure the long-term outcomes of hyperbaric treatment in children with autism, additional studies are needed to determine if the significant improvements observed in this study last beyond the study period. It is possible that ongoing treatments would be necessary to maintain the improvements observed, but this study was not designed to examine that possibility. Our clinical observations in children with autism suggest that additional hyperbaric treatments beyond 40 total sessions can lead to additional improvements; however, further studies are needed to formally validate these observations. Recently, several companies have started producing and marketing portable hyperbaric chambers that are approved by the U.S. Food and Drug Administration (FDA) for home use and are able to supply the hyperbaric treatment parameters used in this study. Therefore, the widespread and long-term use of this potential treatment is feasible and not necessarily costly (on a per treatment basis). Finally, this study was not designed to determine if higher hyperbaric treatment parameters (higher atmospheric pressure and oxygen levels, which can only be provided in a clinic setting) would lead to better or more long-lasting results. Additional studies are needed to investigate that possibility.

## Conclusion

Given the positive findings of this study, and the shortage of proven treatments for individuals with autism, parents who pursue hyperbaric treatment for their child with autism can be assured that it is a safe treatment modality at the pressure used in this study (1.3 atm), and that it may improve certain autistic behaviors. Further studies are needed by other investigators to confirm these findings; we are aware of several other planned or ongoing studies of hyperbaric treatment in children with autism. However, in light of the positive results of this study and those of several previous studies [7,17,21,22,35], the use of hyperbaric treatment appears to be a promising treatment for children with autism.

## Abbreviations

ABA: Applied Behavioral Analysis; ABC: Aberrant Behavior Checklist; ADI-R: Autism Diagnostic Interview-Revised; ADOS: Autism Diagnostic Observation Schedule; ASD: Autism Spectrum Disorder; ATEC: Autism Treatment Evaluation Checklist; atm: atmosphere; CGI: Clinical Global Impression; FDA: Food and Drug Administration; HIF-1α: Hypoxia-Inducible Factor 1α; IHA: International Hyperbarics Association; MRI: Magnetic Resonance Imaging; ns: not significant; PDD-NOS: pervasive developmental disorder-not otherwise specified; PET: Positron Emission Tomography; SPECT: Single Photon Emission Computed Tomography; U.S.: United States; VEGF: Vascular Endothelial Growth Factor.

## Competing interests

DAR, LWR, SS, CS, AU, JN, EMM, and EAM treat individuals with hyperbaric treatment in their clinical practices and derive revenue from hyperbaric treatment. DAR, LWR, and EAM had previously received research funding from the International Hyperbarics Association for an earlier study of hyperbaric treatment in autism [7]. EAM has also received hyperbaric chambers and financial support (unrelated to this study) from OxyHealth LLC for remodeling the Rimland Center, a center for mentoring clinicians interested in learning how to care for children with autism spectrum disorders. The remaining authors (SL, GH, and BG) declare that they have no competing interests.

## Authors' contributions

DAR, LWR, and EAM conceived of the study and the study design. DAR, SS, CS, AU, JN, EMM, and EAM performed initial physical exams on the children and were responsible for completing the physician CGI scales. SL and GH performed psychological testing on the children including ADOS and ADI-R. DAR and BG performed the statistical analysis. All authors read and approved the final manuscript.

## Pre-publication history

The pre-publication history for this paper can be accessed here:



## Supplementary Material

Additional File 1 **Table 2.** Changes on the ABC scale and subscales in the treatment and control groups (lower scores denote improvement). # Wilcoxon signed rank tests. ## Mann Whitney test. Click here for file

Additional File 2 **Table 3.** Changes on the ATEC scale and subscales in the treatment and control groups (lower scores denote improvement). # Wilcoxon signed rank tests. ## Mann Whitney test. Click here for file
