# Supplementary material for: Hyperbaric treatment for children with autism: a multicenter, randomized, double-blind, controlled trial
Source: BMC Pediatr. 2009 Mar 13;9:21. doi: 10.1186/1471-2431-9-21 (PMC2662857; doi:10.1186/1471-2431-9-21)
Supplement: Additional File 1 — Table 2. Changes on the ABC scale and subscales in the treatment and control groups (lower scores denote improvement). # Wilcoxon signed rank tests. ## Mann Whitney test. [file 1471-2431-9-21-S1.doc]

Table 2

| **ABC Scores** | Treatment Group  Pre-treatment | Treatment Group  Post-treatment | % Change | p-value^#^ | Control Group  Pre-treatment | Control  Group  Post-treatment | % Change | p-value^#^ | p-value^##^  between  groups |
| --- | --- | --- | --- | --- | --- | --- | --- | --- | --- |
| Total | 55.2 ± 28.7 | 46.4 ± 24.7 | 16.0 | **0.0118** | 53.3 ± 24.0 | 45.5 ± 17.3 | 14.5 | 0.1140 | 0.4092 |
| Irritability | 13.2 ± 9.5 | 10.5 ± 7.4 | 20.6 | **0.0147** | 12.2 ± 7.9 | 11.3 ± 6.4 | 7.6 | 0.5171 | 0.0976 |
| Social Withdrawal | 10.5 ± 6.9 | 9.3 ± 6.7 | 10.9 | 0.1601 | 11.2 ± 6.9 | 8.9 ± 5.6 | 20.6 | 0.1511 | 0.9766 |
| Stereotypy | 7.5 ± 4.9 | 6.2 ± 5.1 | 17.5 | **0.0124** | 6.2 ± 4.7 | 5.4 ± 4.0 | 13.0 | 0.1454 | 0.3217 |
| Hyperactivity | 20.7 ± 9.9 | 17.8 ± 9.2 | 13.9 | **0.0211** | 20.1 ± 8.2 | 16.8 ± 7.7 | 16.6 | 0.0794 | 0.7404 |
| Speech | 3.4 ± 3.1 | 2.6 ± 2.5 | 23.2 | **0.0155** | 3.6 ± 3.6 | 3.3 ± 3.2 | 7.5 | 0.4263 | 0.2955 |
