# Supplementary material for: Hyperbaric treatment for children with autism: a multicenter, randomized, double-blind, controlled trial
Source: BMC Pediatr. 2009 Mar 13;9:21. doi: 10.1186/1471-2431-9-21 (PMC2662857; doi:10.1186/1471-2431-9-21)
Supplement: Additional File 2 — Table 3. Changes on the ATEC scale and subscales in the treatment and control groups (lower scores denote improvement). # Wilcoxon signed rank tests. ## Mann Whitney test. [file 1471-2431-9-21-S2.doc]

Table 3

| **ATEC Scores** | Treatment Group  Pre-treatment | Treatment Group  Post-treatment | % Change | p-value^#^ | Control Group  Pre-treatment | Control Group  Post-treatment | % Change | p-value^#^ | p-value^##^ between groups |
| --- | --- | --- | --- | --- | --- | --- | --- | --- | --- |
| Total | 75.3 ± 19.5 | 65.9 ± 16.4 | 12.5 | **0.002** | 75.6 ± 21.0 | 70.1 ± 21.9 | 7.2 | **0.0385** | 0.1996 |
| Speech/Language | 16.3 ± 5.0 | 15.5 ± 5.1 | 5.1 | 0.1167 | 15.9 ± 6.1 | 15.4 ± 6.6 | 3.3 | 0.2754 | 0.7754 |
| Sociability | 17.4 ± 6.6 | 14.5 ± 6.5 | 16.5 | **0.0009** | 17.8 ± 6.2 | 16.0 ± 6.8 | 9.7 | **0.0134** | 0.2005 |
| Sensory/Cognitive | 18.1 ± 5.2 | 15.1 ± 3.9 | 16.6 | **0.0017** | 19.6 ± 5.6 | 18.5 ± 6.2 | 5.4 | 0.1439 | **0.0367** |
| Health/Physical | 23.5 ± 11.5 | 20.8 ± 8.7 | 11.3 | **0.0446** | 22.4 ± 8.3 | 20.2 ± 7.3 | 9.8 | 0.1674 | 0.4296 |
